# Supplementary material for: Is it cognitive effort you measure? Comparing three task paradigms to the Need for Cognition scale
Source: PLoS One. 2023 Aug 17;18(8):e0290177. doi: 10.1371/journal.pone.0290177 (PMC10434945; doi:10.1371/journal.pone.0290177)
Supplement: S1 File — (DOCX) [file pone.0290177.s001.docx]

# Supplementary material for: “Is it cognitive effort you measure? Comparing three task paradigms to the Need for Cognition scale”

## Additional information for Study 1

Results: Participants had a demand avoidance (preference for the high demand deck: M = .42, SD = .17) that was not significantly different from indifference between the high and low demand option (Z = 0.674, p < 0.5, d = .077). Accuracy in the DST was high (M = 0.94, SD = 0.07). The COGED paradigm showed significantly larger monetary discounting with increasing load levels F(4, 380) = 24.109, p < .001, η^2^ = .202. The average indifference point was 1.46 (SD = .44). Across all six n-back levels the discriminability d’ was 2.06 (SD = .7) and perceived mental effort was 66 (SD = 18.2) on a scale from 0 to 100. The Need for Cognition Score ranged from 35 to 86, with a mean of 65.1 (SD = 11.3). The average O-span performance was 51.82 (SD = 16.81).

None of the tasks was related to working memory as assessed with the O-Span task, all τ < .01, all p > .2. O-Span and d’ were not associated, τ = .101, p = .2.

## Additional information for Study 2

Results: Like study 1, participants performed well on the O-span (M = 50.78, SD = 18.57), their NCS ranged from 36 to 84 with a mean of 66.56 (SD = 9.82). They had on average over half of the rational reasoning items correct (M = 10.04, SD = 4.04). The COGED paradigm showed significantly larger monetary discounting with increasing load levels, F(4, 240) = 24.734, p < .001, η^2^ = .191. The average indifference point was 1.58 (SD = .42). Across all six n-back levels the discriminability d’ was 1.68 (SD = .76).

O-Span was positively correlated with rational reasoning, τ = .209, p = .008 and d’, τ = .153, p = .045, but not with cognitive effort discounting, τ = .096, p = .226; or Need for Cognition, τ = .006, p = .422.

## Additional information for Study 3

Methods: Regarding the rational reasoning items we used items 2-7 from the Cognitive Reflection Test [1], one fully disjunctive reasoning problem “the marriage problem” [2], one probability matching task [3], one probability estimation task “the bus problem” [4], one making sense of medical results problem [5], one Bayesian reasoning problem [6], one covariation detection problem [7], one knight and knave problem [8], one conditional reasoning problem [9].

Results: The Need for Cognition score ranged from 42 to 81 with a mean of 61.73 (SD = 9.15). The indifference point was M = 1.18 (SD = .56), discriminability d’ was M = 2.08 (SD = .66) and perceived mental demand was high, M = 73.13 (SD = 16.04). The rational reasoning score was M = 5.08 (SD = 2.49) and perceived mental demand was M = 67.2 (SD = 14.95).

## Additional information for Study 4

Methods: Filler tasks in study 4: On day 1 participants did also the Bullshit receptivity scale [10]), the Effort expenditure for rewards task (EEfRT, [11]) and N-TLX_EEfRT_. On Day 2 they did after the DST and NCS a Handgrip task [12]

Results: For the Demand Selection Task, accuracy was high on Day 1 (M = 0.98, SD = 0.02) and Day 2 (M = 0.96, SD = 0.08). The median of high demand choice was 0.49 for Day 1 and Day 2. Demand avoidance was not different from .5, neither on day Day 1 (Z = .21, p = .834, d = .034) nor on Day 2 (Z = .238, p = .812, d = .039). Debriefing identified 5 participants on Day 1 that identified the manipulation (two types of decks) and 11 participants who might have. On Day 2 the manipulation was found by 13 participants, and another 22 might have found it. Among those who discovered the manipulation the high demand avoidance was .6 on Day 1 and .54 on Day 2. One participant who noticed the manipulation said they tried not to exploit it. On Day 2 demand avoidance was not related to NCS (τ = .165, p = .16).

The COGED paradigm showed significant increases in monetary discounting with increasing load levels, F(2, 114) = 4.432, p = .014, η^2^ = .072. The average indifference point was 1.16 (SD = .51) and discriminability d’ had M = 2.37 (SD = .3). Participants had on average half of the rational reasoning items correct, M = 7.0 (SD = 2.7). The Need for Cognition score had M = 62 (SD = 10.11) on day 1 and M = 65 (SD = 11.5) on day 2. Need for Cognition had good internal consistency on Day 1 (Cronbach’s α = .83) and Day 2 (Cronbach’s α = .89), and good reliability across the two testing sessions (r = .823, p < .001).

## Additional information for Study 5

The Need for Cognition score ranged from 33 to 83, M = 63.46 (SD = 9.77). On average participants solved half of the items correctly, M = 6.82 (SD = 2.61) and perceived mental demand was high, M = 79.11 (SD = 18.87). Accuracy on the demand selection task was high, M = 0.97 (SD = 0.04), perceived mental demand low, M = 45.56 (SD = 24.5). Participants avoided cognitive demand, M = .45 (SD = .12, Md = 0.47) but demand preference was not significantly different from chance (Z test, Z = .37, p < 0.711, d = .055).

## Additional information for Study 6

Methods: Regarding the rational reasoning items we used items 2-7 from the Cognitive Reflection Test [1], one fully disjunctive reasoning problem, “the marriage problem” [2], one knight and knave problem [8], one conditional reasoning problem [9], one covariation problem [13], one base rate problem [14], one making sense of medical results problem [5].

Results: The two samples had similar NCS scores, t(197.38) = .838, p = .403, M_pooled_ = 61.59, SD = 10.94, similar working memory capacity, t(203.32) = -.12, p = .904, M_pooled_ = 3.31, SD = 2.13, similar accuracy in the DST, t(208.31) = -.062, p = .95, M_pooled_ = .92, SD = .076 and similar demand avoidance, t(127.77) = 1.074, p = .285, M_pooled_ = .44, SD = .23. The rational reasoning score was higher in the UiT sample (M = 6.01, SD = 2.71) compared to the Prolific sample (M = 4.83, SD = 2.62). This difference was significant; t(163.75) = -3.633, p = .00037). The average indifference point in COGED was higher in the Prolific sample (M = 1.24, SD = .57) than in the UiT sample (M = 1.01, SD = .58). This difference was significant, t(164.97) = 3.157, p = .002).

## Differences between the six studies

We expressed the rational reasoning score as percentage correct. A one-way ANOVA yielded a significant difference between studies (F(4, 577) = 11.253, p < .001, η^2^ = .072). Post-hoc Tukey HSD found a significant difference between study 2 and 3 (t = 6.389, p < .001), study 2 and 6 (t = 5.108, p < .001), study 3 and 4 (t = 2.741, p = .049), study 3 and 5 (t = 3.271, p = .01) and study 3 and 6 (t = 2.786, p = .044). Since the total number of items differed across studies, we z-scored the values.

One-way ANOVA yielded a significant difference between studies for cognitive effort discounting (indifference point) in the COGED (F(4, 585) = 12.683, p < .001, η^2^ = .08. Post-hoc Tukey HSD test was significant for study 1 versus study 3 (t = 3.571, p = .004) and study 1 versus study 6 (t = 4.372, p < .001), study 2 differed from study 3 (t = 4.869, p < .001), study 4 (t = 2.817, p = .04) and study 6 (t = 6.05, p < .001). Since study 1 and 2 used up to 6-back whereas studies 3-6 used only up to 4-back, we z-scored the indifference point values.

One-way ANOVA yielded no significant difference between studies for the DST (F(3, 472) = .386, p = .763, η^2^ = .002). The DST was significantly below .5 (M = .44, SD = .2), i.e., on average participants avoided the high demand option (t(475) = 6.555, p < .001, Cohen’s d= .3).

One-way ANOVA yielded a significant difference between studies for NCS (F(5, 634) = 4.058, p < .001, η^2^ = .031). Post-hoc Tukey HSD test was significant for the comparison of study 2 with study 3 (t = 3.084, p = .026) and study 2 with study 6 (t = 3.96, p = .001). No other comparison was significant (see SOM for details).

One-way ANOVA yielded a significant difference between studies for d’ (F(4, 588) = 19.281, p < .001, η^2^ = .116). Post-hoc Tukey HSD test was significant for the comparison of study 1 with study 6 (t = 4.467, p < .001), study 2 with study 3 (t = 2.849, p = .037), study 2 with study 6 (t = 7.428, p < .001), study 3 with study 6 (t = 3.887, p < .001) and study 4 with study 6 (t = 3.136, p = .015).

## Study-wise bi-variate scatterplots

### Figure S1: Need for Cognition Score (NCS) and d’ (dprime) from n-back phase from COGED


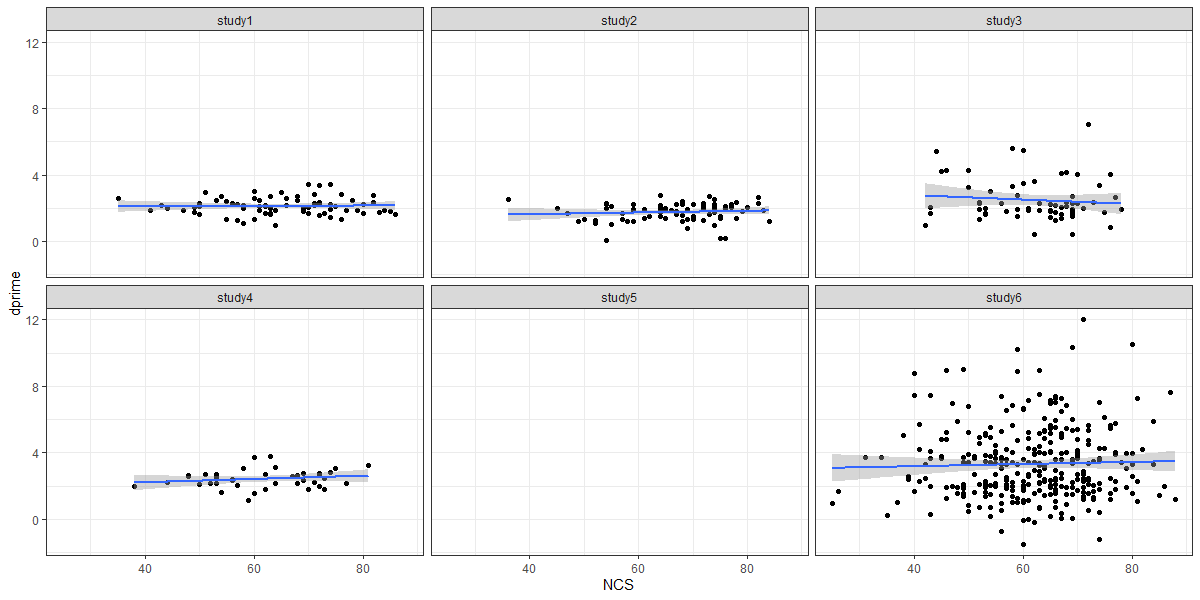


### Figure S2: Need for Cognition Score (NCS) and average indifference point from COGED


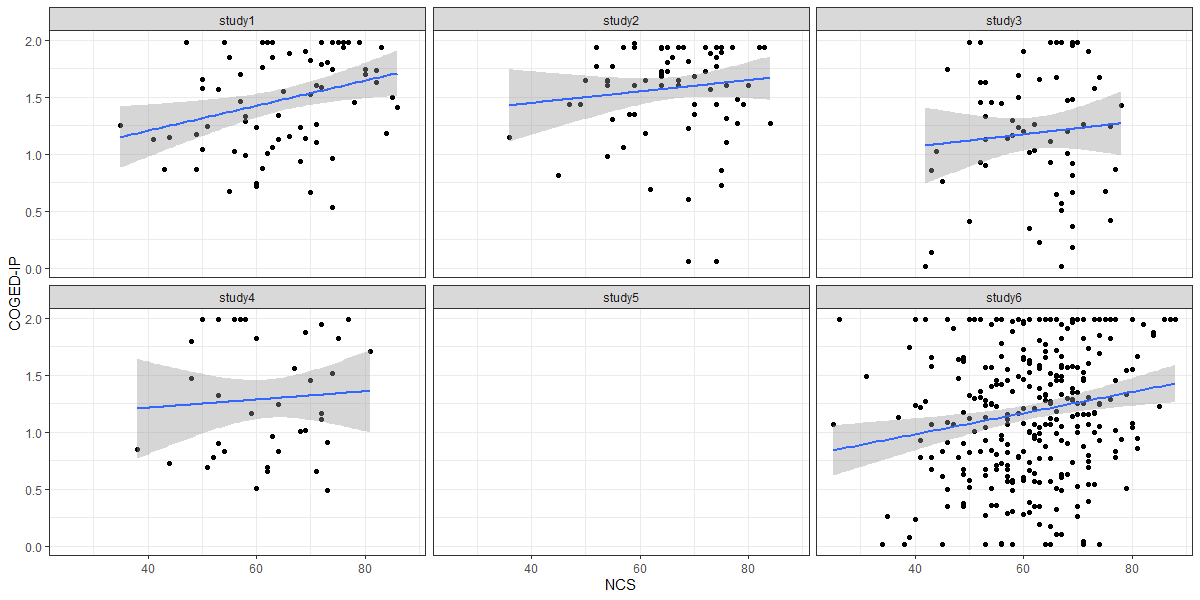


### Figure S3: Need for Cognition Score (NCS) and proportions high demand choices in DST


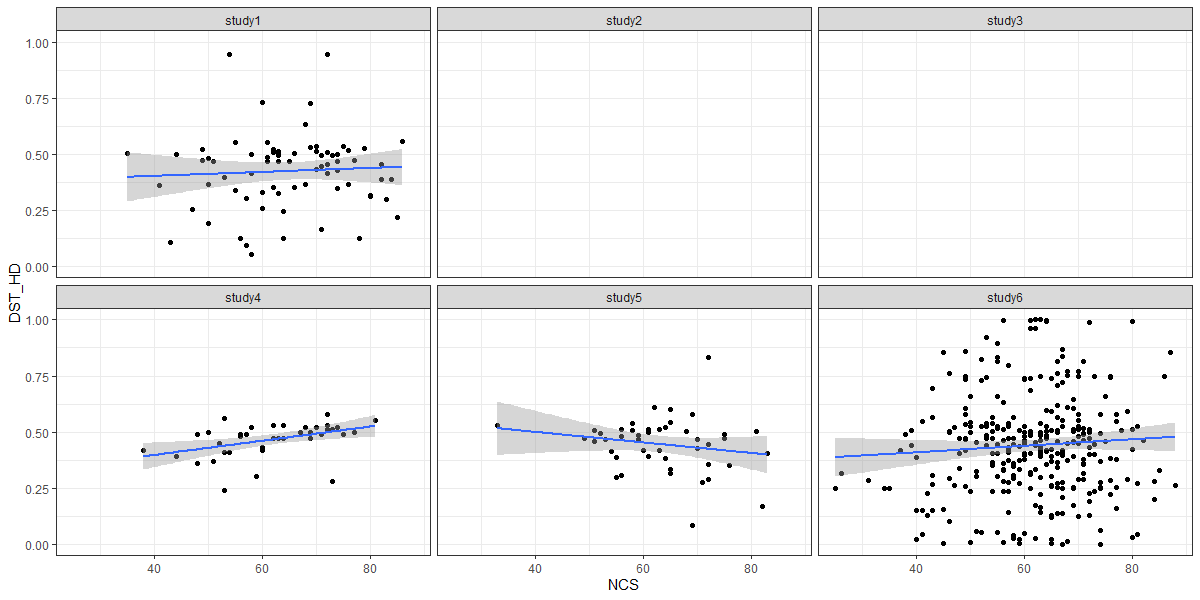


### Figure S4: Need for Cognition Score (NCS) and rational reasoning score


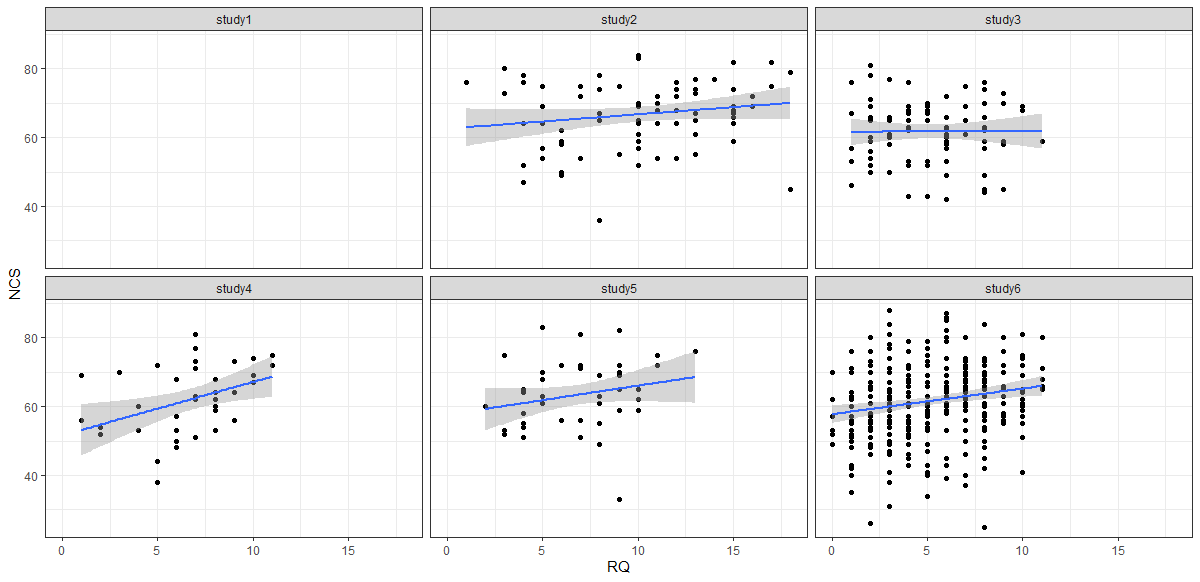


### Figure S5 d‘ and indifference point from COGED


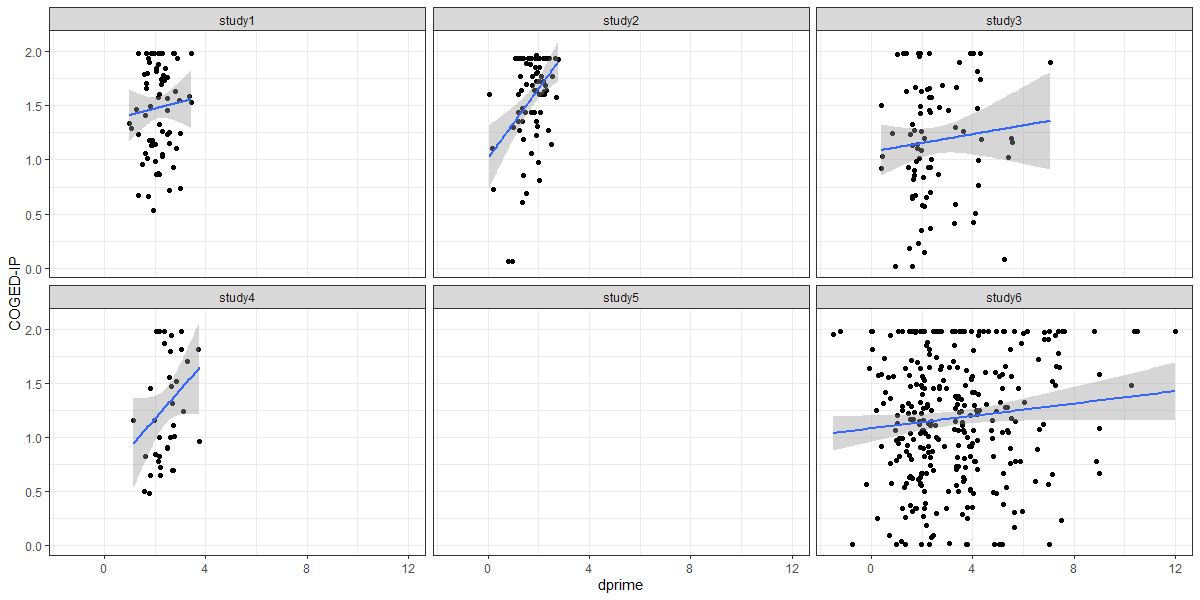


### Figure S6 d‘ and proportion high demand choices in DST


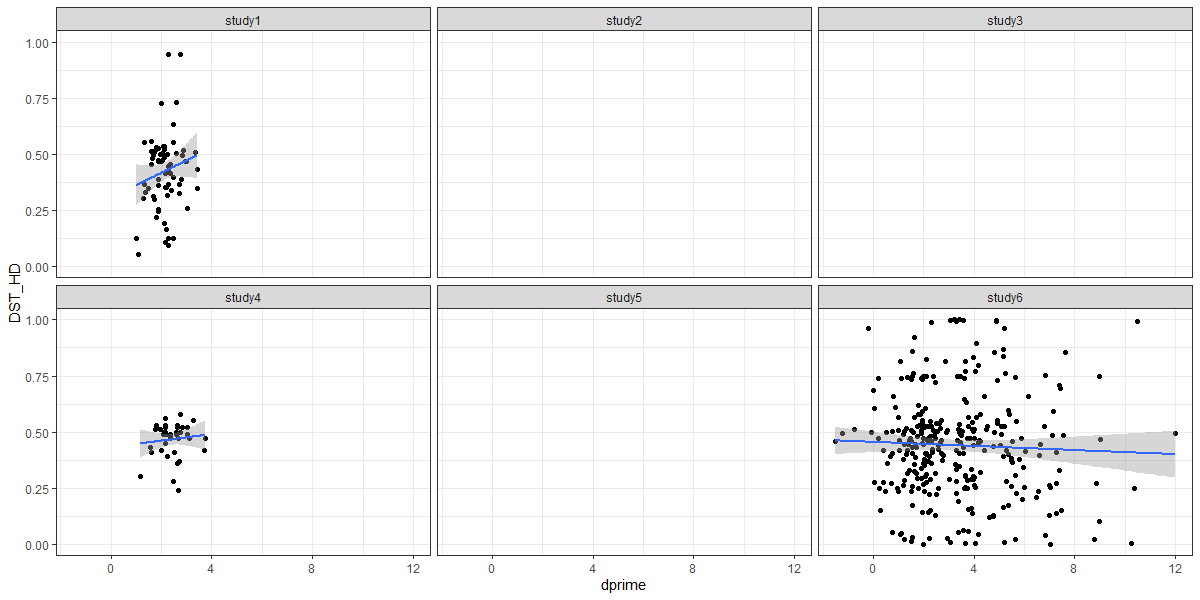


### Figure S7 d‘ and rational reasoning score
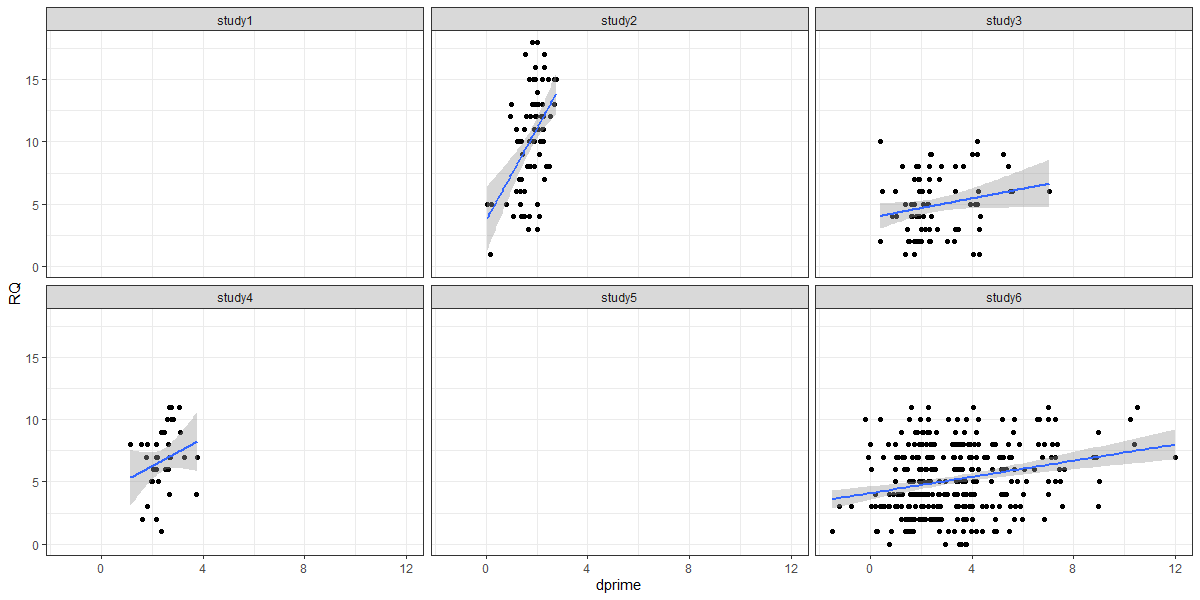


### Figure S8 indifference point from COGED and proportion high demand choices in DST


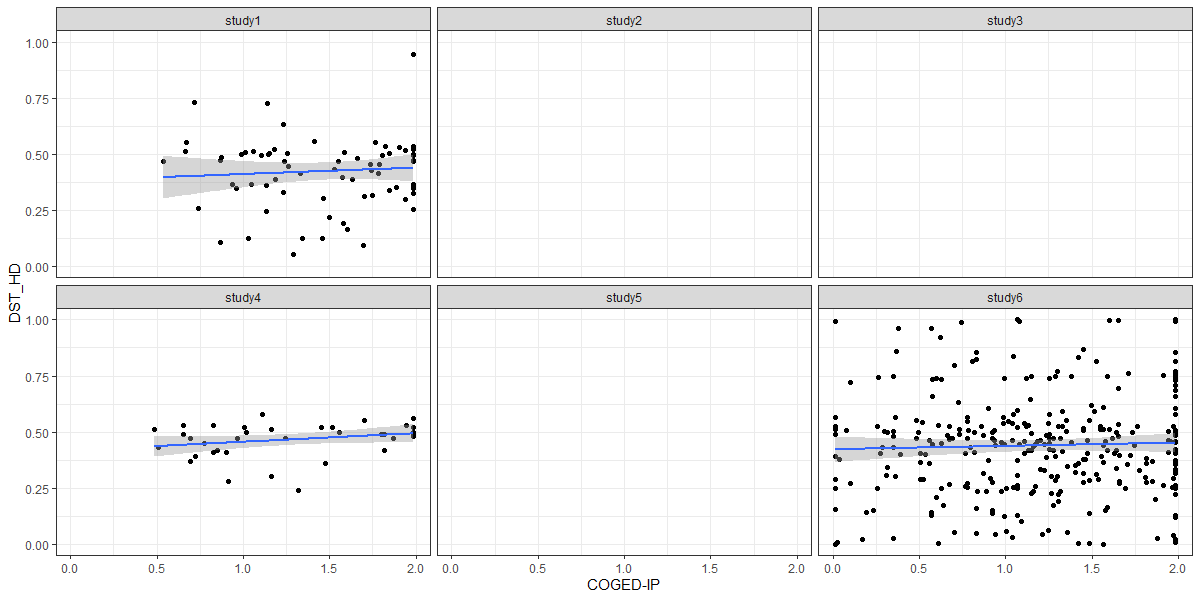


### Figure S9 indifference point from COGED and rational reasoning score


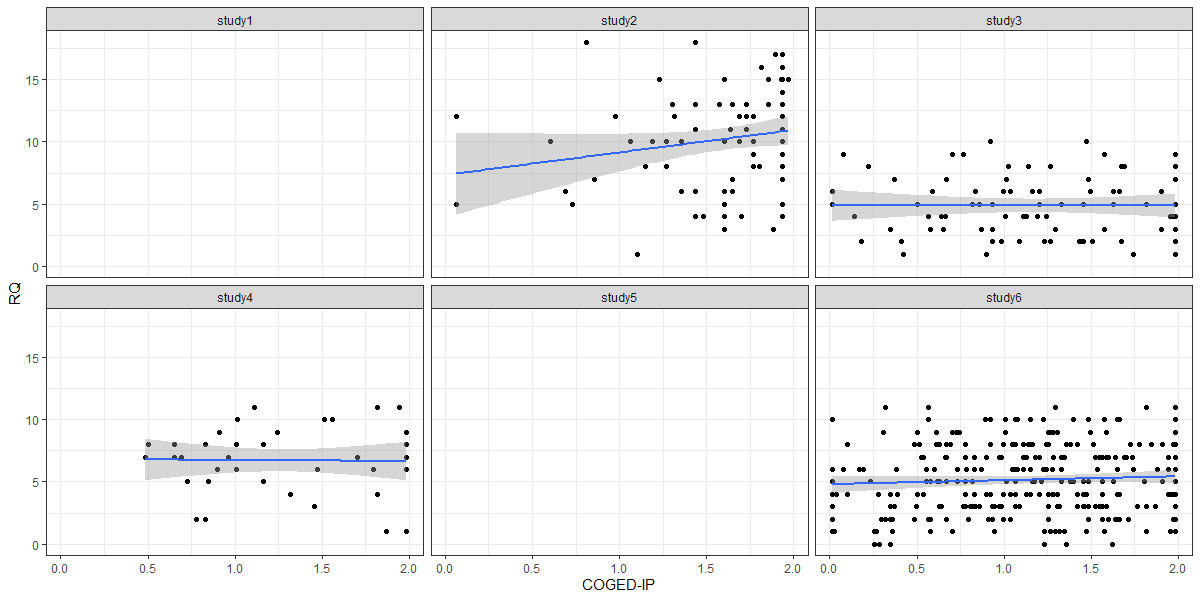


### Figure S10 proportion high demand choices in DST and rational reasoning score


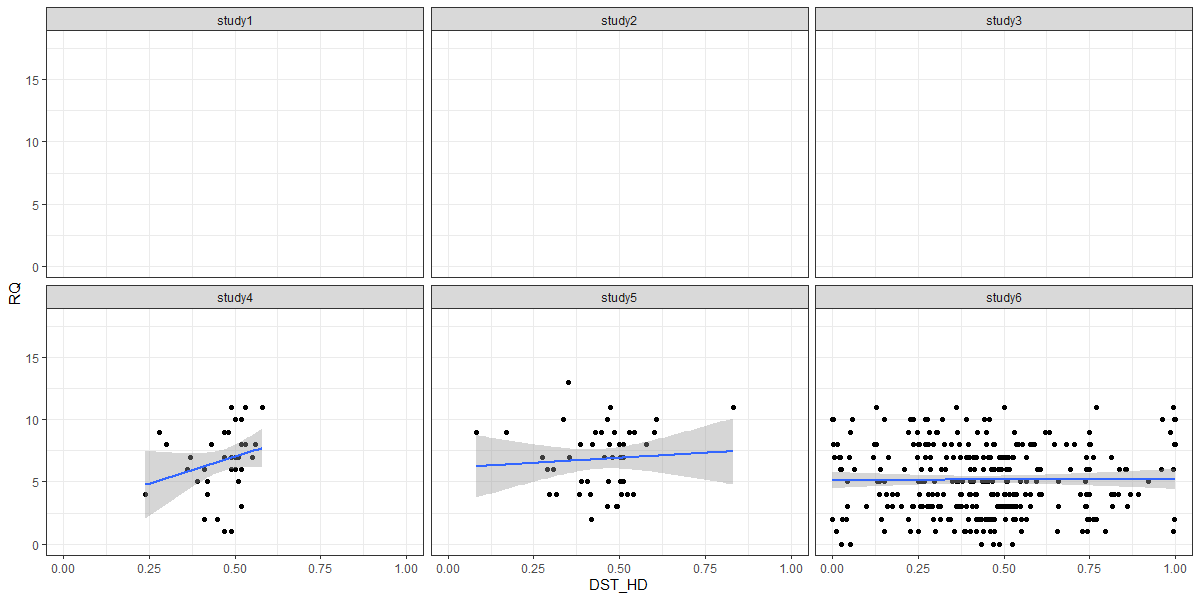


## Alternative analysis: Pooling after z-scoring

We z-scored all values per study to compare across studies. The Pearson product-moment correlations with p-value and Bayes Factor (BF) are shown in Table 1. BF_10_ < .3 provides support for an absence of a relationship between the tasks. BF_10_ > 3 provides support for the hypothesis that there is an association between the tasks.

| **Table S1.** Pearson's Correlations and Bayes Factors | | | | | | | | | | | | | | | | | | | |  |  |
| --- | --- | --- | --- | --- | --- | --- | --- | --- | --- | --- | --- | --- | --- | --- | --- | --- | --- | --- | --- | --- | --- |
|  | |  |  | | | n | | | Pearson's r | | | p | | | Lower  95% CI | | | Upper  95% CI | | BF_10_ |  |
| Effort discounting, AIP |  | - |  | Demand Avoidance |  | | 430 |  | | 0.0669 |  | | 0.1661 |  | | -0.0278 |  | | 0.1605 | **0.1571** |  |
| Effort discounting, AIP |  | - |  | Rational reasoning score |  | | 516 |  | | 0.0535 |  | | 0.2254 |  | | -0.0330 |  | | 0.1391 | **0.1147** |  |
| Effort discounting, AIP |  | - |  | Need for Cognition score |  | | 577 |  | | 0.1769 |  | | < .001 |  | | 0.0967 |  | | 0.2549 | 473.0110 |  |
| Effort discounting, AIP |  | - |  | n-back d’ |  | | 591 |  | | 0.1092 |  | | 0.0079 |  | | 0.0288 |  | | 0.1882 | 1.7421 |  |
| Demand Avoidance |  | - |  | Rational reasoning score |  | | 402 |  | | 0.0326 |  | | 0.5146 |  | | -0.0654 |  | | 0.1300 | **0.0772** |  |
| Demand Avoidance |  | - |  | Need for Cognition score |  | | 478 |  | | 0.0692 |  | | 0.1310 |  | | -0.0206 |  | | 0.1579 | **0.1786** |  |
| Demand Avoidance |  | - |  | n-back d’ |  | | 431 |  | | 0.0067 |  | | 0.8897 |  | | -0.0878 |  | | 0.1011 | **0.0609** |  |
| Rational reasoning score |  | - |  | Need for Cognition score |  | | 568 |  | | 0.1768 |  | | < .001 |  | | 0.0960 |  | | 0.2554 | 409.2896 |  |
| Rational reasoning score |  | - |  | n-back d’ |  | | 519 |  | | 0.2736 |  | | < .001 |  | | 0.1921 |  | | 0.3514 | 2.7843e +7 |  |
| Need for Cognition score |  | - |  | n-back d’ |  | | 579 |  | | 0.0420 |  | | 0.3126 |  | | -0.0396 |  | | 0.1231 | **0.0866** |  |
|  | | | | | | | | | | | | | | | | | | | |  |  |

Legend: BF_10_ in bold support the null hypothesis, BF_10_ in italic support the alternative hypothesis. P value in italic support the alternative hypothesis

There are no differences to the meta-analytical approach regarding significance. This analysis complements the analysis reported in the main text by providing Bayes Factors and hence support for the null hypothesis.

## Lab vs online studies

The demand selection task yielded similar demand avoidance in lab and online samples, t(456.017) = .049, p = .961, Cohen’s d = .004. The Need for Cognition score was higher among lab- than online participants, t(633.279) = 2.878, p = .004, Cohen’s d = .228. Working memory capacity was higher in the online than the lab samples, t(434.607) = -8.64, p < .001, Cohen’s d = .693. The average indifference point was higher in the lab than online sample, t(587.977) = 4.784, p < .001, Cohen’s d = .393. Rational reasoning score (percentage correct) was similar in the lab and the online sample, t(580) = 1.715, p = .087, Cohen’s d = .143.

## Study S1: Test – re-test reliability of rationality items

## Methods

### Ethics

All methods were performed in accordance with the relevant guidelines and regulations and approved by the Institutional Review Board at the Department of Psychology at UiT – The Arctic University of Norway. Written informed consent was obtained from all participants.

### Participants

In total 136 participants were recruited at Prolific (prolific.co). Of the 136, 83 completed both sessions and all questionnaires and had sufficient proficiency in English (assessed with the Word sum test, cut-off was 3 out of 10). 36 of the participants were women (aged 18- 49) and 46 were men (aged 18-64), one indicated as gender other. The experiment was conducted online, allowing people from several countries to participate (24% from Poland, 13.5% Portugal, 12.5% Italy, 10% England, remaining were from 13 other countries). Participants received ca. £10 pounds after completion. Participation was voluntary and participants could withdraw their consent at any moment.

### Materials

The experiment was conducted in English and all instructions were in English.

Regarding rational reasoning items we used 14 items from the problem solving and reasoning literature:

- One item from [15].
- One item from [16].
- Items two and three from [17].
- Items 4-6 from [18].
- One item from [2].
- One item from [19].
- One item from [14].
- One item from [20].
- One item from [8]
- One item from the Wason selection task (Wason, 1966; as cited in [21]).
- One item from [22].

Although the degree of difficulty varied in these items, deliberate reasoning was required in all to reach the correct answer. The 14 items were sequentially and randomly presented to all participants. Participants had to provide an answer before proceeding to the next item. There were no time limits for answering the RQ items in session 1.

In session 1 we measured also cognitive abilities with the Berlin numeracy test [23] and the word sum test [24], as well as we used the Need for Cognition scale. These questionnaires are not of interest here. We also used the NASA Task load index.

The 14 items were divided into two sets after session 1 (based on response times). Set 1 were; both items from Thomson and Oppenheimer, one from Finucane and Gullion, the item from Wason and Brooks, the item from Kahneman and Tversky, the item from Wason, and the item from Shafir. The remaining seven items made up set 2.

Approximately three weeks after session 1 participants got either set 1 or 2 again (after having played the Dice task, an information sampling task, not of interest here).

The study was administered in Qualtrics (Qualtrics, Provo, UT).

### Analysis

The test – retest score is based on the performance of the 7 items in session 1 that are identical with the items received in session 2.

## Results

Participants got on average 49% of the items correct in session 1 and 50% in session 2. Test – retest correlation was Pearson’s ρ = .701, p < .001. Test - retest correlation between all 14 items in session 1 and the seven items in session 2 was ρ = .8809, p < .001.

## Study S2: Test – retest reliability of the Cognitive Effort discounting task

## Methods

### Ethics

All methods were performed in accordance with the relevant guidelines and regulations and approved by the Institutional Review Board at the Department of Psychology at UiT – The Arctic University of Norway. Written informed consent was obtained from all participants.

### Participants

In total 25 participants were recruited, and all completed all three sessions (10 women, 15 men, aged 19-31 years). Inclusion criteria were good health, tolerance for caffeine, sucrose or artificial sweetener as this study served as pilot for a study on the effect of energy drinks on cognitive ability and effort. The participants were compensated for their time with a gift card at their local grocery store valued at 400 NOK (approximately $40).

### Materials

The experiment was conducted in English and all instructions were in English. We used the COGED as in experiment 6 with two modifications. In the training phase participants filled out the N-TLX after each n-back level. The instruction slides had a fixed time to ensure that all spent a similar amount of time on the instructions. COGED testing lasted approximately 35 min.

### Analysis

We averaged the indifference point (IP) for the choice options 1- vs 2-back, 1- vs-3-back and 1- vs 4-back. Correlation between the averaged IP from session 1 and 2, and session 2 and 3 were calculated.

## Results

The average IP was 1.41 in session 1, 1.57 in session 2 and 1.74 in session 3. Participants choose higher n-back levels the more proficient they got with the task. Test – retest correlation was high, Pearson’s ρ = .789 for session 1 with session 2, and ρ = .819 for session 2 with session 3.

## Additional references

1. Toplak ME, West RF, Stanovich KE. Assessing miserly information processing: An expansion of the Cognitive Reflection Test. Thinking & Reasoning. 2014;20(2):147-68. doi: 10.1080/13546783.2013.844729.

2. Levesque HJ. Knowledge Representation and Reasoning. Annual Review of Computer Science. 1986;1(1):255-87. doi: 10.1146/annurev.cs.01.060186.001351.

3. Koehler DJ, James G. Probability matching and strategy availability. Mem Cognit. 2010;38(6):667-76. Epub 2010/09/21. doi: 10.3758/mc.38.6.667. PubMed PMID: 20852231.

4. Teigen KH, Keren G. Waiting for the bus: when base-rates refuse to be neglected. Cognition. 2007;103(3):337-57. Epub 2006/05/26. doi: 10.1016/j.cognition.2006.03.007. PubMed PMID: 16723123.

5. Gigerenzer G, Gaissmaier W, Kurz-Milcke E, Schwartz LM, Woloshin S. Helping Doctors and Patients Make Sense of Health Statistics. Psychol Sci Public Interest. 2007;8(2):53-96. Epub 2007/11/01. doi: 10.1111/j.1539-6053.2008.00033.x. PubMed PMID: 26161749.

6. Toplak ME, Liu E, Macpherson R, Toneatto T, Stanovich KE. The reasoning skills and thinking dispositions of problem gamblers: A dual-process taxonomy. Journal of Behavioral Decision Making. 2007;20(2):103-24. doi: 10.1002/bdm.544.

7. Stanovich KE, West RF. Individual differences in rational thought. Journal of Experimental Psychology: General. 1998;127(2):161-88. doi: 10.1037/0096-3445.127.2.161.

8. Smullyan RM. What is the name of this book? The riddle of Dracula and other logical puzzles. Englewood Cliffs, NJ: Prentice-Hall; 1978.

9. Lehman DR, Lempert RO, Nisbett RE. The effects of graduate training on reasoning: Formal discipline and thinking about everyday-life events. American Psychologist. 1988;43(6):431-42. doi: 10.1037/0003-066X.43.6.431.

10. Pennycook G, Allan Cheyne J, Barr N, Koehler DJ, Fugelsang JA. On the reception and detection of pseudo-profound bullshit. Judgment and Decision Making. 2015;10(6):549-63. Epub 2023/01/01. doi: 10.1017/S1930297500006999.

11. Treadway MT, Buckholtz JW, Schwartzman AN, Lambert WE, Zald DH. Worth the 'EEfRT'? The effort expenditure for rewards task as an objective measure of motivation and anhedonia. PLoS One. 2009;4(8):e6598. Epub 2009/08/13. doi: 10.1371/journal.pone.0006598. PubMed PMID: 19672310; PubMed Central PMCID: PMCPMC2720457.

12. Xu X, Demos KE, Leahey TM, Hart CN, Trautvetter J, Coward P, et al. Failure to Replicate Depletion of Self-Control. PLOS ONE. 2014;9(10):e109950. doi: 10.1371/journal.pone.0109950.

13. Toplak ME, West RF, Stanovich KE. The Cognitive Reflection Test as a predictor of performance on heuristics-and-biases tasks. Memory & Cognition. 2011;39(7):1275. doi: 10.3758/s13421-011-0104-1.

14. West RF, Toplak ME, Stanovich KE. Heuristics and Biases as Measures of Critical Thinking: Associations with Cognitive Ability and Thinking Dispositions. Journal of Educational Psychology. 2008;100(4):930-41. doi: 10.1037/a0012842.

15. Van Dooren W, De Bock D, Evers M, Verschaffel L. Students' Overuse of Proportionality on Missing-Value Problems: How Numbers May Change Solutions. Journal for Research in Mathematics Education. 2009;40(2):187-211.

16. Primi C, Morsanyi K, Chiesi F, Donati MA, Hamilton J. The Development and Testing of a New Version of the Cognitive Reflection Test Applying Item Response Theory (IRT). Journal of Behavioral Decision Making. 2016;29(5):453-69. doi: <https://doi.org/10.1002/bdm.1883>.

17. Thomson KS, Oppenheimer DM. Investigating an alternate form of the cognitive reflection test. Judgment and Decision Making. 2016;11(1):99-113.

18. Finucane ML, Gullion CM. Developing a tool for measuring the decision-making competence of older adults. Psychology and aging. 2010;25(2):271-88. doi: 10.1037/a0019106. PubMed PMID: 20545413; PubMed Central PMCID: PMCPMC2918639.

19. Wason PC, Brooks PG. THOG: The anatomy of a problem. Psychological Research. 1979;41(1):79-90. doi: 10.1007/BF00309425.

20. Kahneman D, Tversky A. Subjective probability: A judgment of representativeness. Cognitive Psychology. 1972;3(3):430-54. doi: <https://doi.org/10.1016/0010-0285(72)90016-3>.

21. Kornreich C, Delle-Vigne D, Brevers D, Tecco J, Campanella S, Noël X, et al. Conditional Reasoning in Schizophrenic Patients. Evolutionary Psychology. 2017;15(3):1474704917721713. doi: 10.1177/1474704917721713. PubMed PMID: 28783973.

22. Shafir E. Uncertainty and the difficulty of thinking through disjunctions. Cognition. 1994;50(1):403-30. doi: <https://doi.org/10.1016/0010-0277(94)90038-8>.

23. Cokely ET, Galesic M, Schulz E, Ghazal S, Garcia-Retamero R. Measuring risk literacy: the berlin numeracy test. Judgment and Decision making. 2012.

24. Cor MK, Haertel E, Krosnick JA, Malhotra N. Improving ability measurement in surveys by following the principles of IRT: The Wordsum vocabulary test in the General Social Survey. Social Science Research. 2012;41(5):1003-16. doi: <https://doi.org/10.1016/j.ssresearch.2012.05.007>.
